# ResNet50-SCPA-net for liver cirrhosis stage classification using MRI images

**DOI:** 10.3389/frai.2026.1881033

**Published:** 2026-08-26

**Authors:** Monisha Perumal, Jagadeesh Gopal

**Affiliations:** School of Computer Science Engineering and Information Systems, Vellore Institute of Technology (VIT), Vellore, Tamil Nadu, India

**Keywords:** CBAM spatial attention, grad-CAM visualization, learnable fusion, parallel structure, SE channel attention

## Abstract

**Introduction:**

Liver cirrhosis is a chronic liver disease that develops in different stages and may cause serious complications if not diagnosed early. However, identifying the stage of liver cirrhosis from MRI images is difficult because the appearance of the liver changes as the disease progresses.

**Methods:**

In this study, a modified ResNet50-based model, called ResNet50-SCPA-Net, was developed for liver cirrhosis stage classification using liver MRI images. The proposed model combined SE channel attention and CBAM spatial attention in a parallel structure to refine the extracted features. A learnable fusion parameter was used to combine the attention features during training. To evaluate the proposed framework, a comparative analysis, an ablation study, and 5-fold cross-validation were performed. Grad-CAM visualization was applied to identify the image regions involved in the prediction process.

**Results:**

The experimental results showed that the proposed model achieved an accuracy of 80.82%, a balanced accuracy of 81.93%, an F1-score of 82.55%, and an AUC of 93.05%. The 5-fold cross-validation produced a mean accuracy of 71.54%, balanced accuracy of 73.57%, and F1-score of 73.13%.

**Conclusion:**

Overall, the findings suggest that the proposed framework can be used to automate liver cirrhosis stage classification from MRI images. Future studies should focus on larger datasets and additional imaging modalities for further evaluation.

## 1 Introduction

The human liver is the largest internal organ and plays a critical role in maintaining good health. Liver disease is a significant health issue worldwide and manifests in various forms. Fatty liver disease, hepatitis, cirrhosis, liver cancer, and chronic liver disease (CLD) are common liver diseases. Among all liver diseases, cirrhosis is the most critical and life-threatening form of liver disease. Cirrhosis has a significant impact on patients’ lives and is considered the final stage of liver disease (Shaheen et al., 2023). However, the incidence of liver disease has rapidly increased over the past few decades and has become one of the main causes of death globally. The World Health Organization (WHO) states that 59% of global mortality and 46% of global diseases are caused by chronic diseases. It also states that 35 million people die each year because of chronic diseases (Shaban, 2024). Fatty liver is characterized by macrovesicular changes without swelling, known as steatosis, and lobular swelling without significant alcohol use (Antunes et al., 2023). Liver fibrosis is characterized by increased deposition of extracellular matrix in response to chronic liver damage, which may lead to cirrhosis and even life-threatening conditions such as hepatocellular carcinoma and liver failure (Gupta et al., 2025). Liver cirrhosis is considered an advanced stage of chronic liver disease, which is one of the major health problems worldwide. This is because cirrhosis is associated with many serious complications related to portal hypertension, including variceal bleeding, ascites, and hepatic encephalopathy (Nowak et al., 2021). Presently, cirrhosis is among the ninth leading cause of death in Europe and Southeast Asia, the fifth in the Eastern Mediterranean region, and the tenth in Africa. Cirrhosis is also one of the top health burdens worldwide in terms of disability-adjusted life years (DALYs). It ranks 15th in terms of DALYs caused by disease burden. The number of DALYs may be even higher in Europe than in Asia (Savaş, 2025). Liver cirrhosis of Child-Pugh Class A implies mild liver disease and the best prognosis of all the other classes (Class B is moderate liver disease, and Class C is severe liver disease; Wu et al., 2021). Medical imaging has become an important tool for assessing liver disease. In the field of liver disease, magnetic resonance imaging (MRI) has become the standard method of liver disease examination because of its advantages, including the lack of ionizing radiation and the use of multiple parameters, multiple directions, multiple sequences, and high-resolution images. The sensitivity of the images obtained using the proposed method for the diagnosis of cirrhosis was higher than that of ultrasound and computed tomography (CT) images (Chunmei et al., 2020). A method that can automatically evaluate relevant characteristics in radiological images can assist radiologists in diagnosing liver cirrhosis, resulting in a more accurate diagnosis and minimal difference in the performance of the reader. In particular, since 2012, when a deep learning method outperformed all other methods with the best performance in the well-known ImageNet competition. Convolutional Neural Networks (CNNs) are considered state-of-the-art models for classifying and segmenting images. In recent years, deep learning techniques have been improved and used effectively in many different areas, including medical research (Nowak et al., 2021). Lin, F. et al. developed a sweetener identification model using a pretrained ResNet50 network with attention mechanism. The SE attention and CBAM were integrated into the ResNet50 architecture to improve feature learning. The model performance was evaluated using both hold-out testing and 5-fold cross-validation approaches (Lin et al., 2024). Prakash, N. N., et al. proposed a DenseNet-based CNN for the automatic prediction and classification of liver lesions. The images were preprocessed using noise removal, image normalization, and contrast enhancement. The images were then segmented to obtain the liver regions. DenseNet was used for feature extraction. The obtained features were classified as benign or malignant lesions. High accuracy was achieved using the proposed method. The method was found to have better accuracy than a traditional CNN (Prakash et al., 2023). Zheng, T., et al. proposed a fully automated CNN-based framework for the noninvasive diagnosis of liver cirrhosis. The features of the images were learned automatically without manual learning. The images were classified as cirrhotic or non-cirrhotic. High accuracy was achieved using the proposed method (Zheng et al., 2024). Table 1 represents the comparison of existing methods for liver cirrhosis classification. Although deep learning has significantly advanced liver disease classification, identifying different stages of cirrhosis remains challenging because of the minimal visual differences between classes. Conventional attention mechanisms generally employ either channel attention or spatial attention independently, or combine both in a fixed sequential manner.

**Table 1:** Comparison of existing methods for liver cirrhosis classification.

Ref	Year	Method	Result	Limitations
Xie et al. (2025)	2025	Triple-Modal Fusion liver cirrhosis network (TMF-LCNet)	Training Cohort *n* = 184, AUC = 0.797; External Test Cohort *n* = 59, AUC = 0.747.	The size of the sample was relatively small.
Gupta et al. (2025)	2025	Two-stage deep learning pipeline: nnU-Net for segmentation and Masked Attention ResNet model for classification.	Performance metrics include a sensitivity of 0.778, specificity of 0.800, an AUC of 0.811, and an accuracy of 0.783.	Performs binary classification of fibrosis severity
Lilhore et al. (2026)	2025	EfficientNet-B7 with dual-attention.	AUC 0.98, accuracy 98.5	More computational time
He et al. (2025)	2025	Hybrid network CMT-Net.	AUC 90, accuracy 86.67.	May miss complementary diagnostic information.
Zang et al. (2023)	2023	SE-ResUnet.	Precision 0.907, recall 0.945.	Required a large amount of annotated data.
Park et al. (2024)	2024	VGGNet, ResNet, DenseNet, EfficientNet and ViT.	The AUC values for VGGNet, ResNet, DenseNet, EfficientNet, and ViT are 0.96, 0.96, 0.95, 0.96, and 0.95, respectively.	May lose fine texture information due to image resizing.
Wu et al. (2021)	2020	ULBP-GLCMfeature+SVMclassifier	Normal liver-100% accuracy Early cirrhosis 100% accuracy, Advanced cirrhosis-99.5.	Fixed ROI size can lead to the loss of important features.
Nowak et al. (2021)	2021	CNN	Accuracy 0.99.	Trained for the identification of liver cirrhosis only.
Hirata et al. (2023)	2023	echo-envelope CNN	Binary accuracy ≥F2, ≥ F3, and ≥ F4 correspond to the accuracies of 91, 80, and 78%, respectively.	Limited dataset
Shaheen et al. (2023)	2023	HCNN-CN-AEPO.	Accuracy 0.993.	Training time is large.
Gaber et al. (2022)	2022	Voting-based ensemble classifier	Accuracy 95.71%	Limited dataset, low quality images
Xue et al. (2020)	2020	Deep convolutional neural network with TL	The AUCs for classification of S4, ≥ S3, and ≥ S2 are 0.950, 0.932, and 0.930, respectively.	Uneven distribution for s4
Tang et al. (2020)	2020	Faster R-CNN, DeepLab	ASD 1.37 ± 0.41	The computational costs are high and require a lot of memory for processing.
Lee et al. (2020)	2020	DCNN	AUC 0.901	Insufficient F2 samples, cannot accurately separate F2 from F3
Alici-Karaca et al. (2022)	2023	Patch-based lightweight convolution neural network	100% for binary classification and 87.57% for multi-class classification	The model performs well for binary classification but shows reduced performance in multi-class classification.
Shaban (2024)	2024	Improved Binary Butterfly Optimization Algorithm	Accuracy 99.1%	Improve computational complexity

When SE attention is integrated with the complete CBAM module, channel recalibration is performed twice because both mechanisms include channel attention, which may introduce redundant feature weighting and additional computational overhead. Moreover, fixed sequential attention does not allow the network to adaptively balance the relative importance of channel and spatial information across different MRI samples. As liver cirrhosis progression is characterized by subtle intensity variations and complex spatial structural changes, effective feature modeling requires complementary rather than redundant attention refinement. Therefore, the proposed ResNet50-SCPA-Net introduces a Parallel Attention Fusion (PAF) module that employs SE exclusively for channel attention while retaining only the spatial attention branch of CBAM. Both attention branches operate in parallel and are adaptively integrated using a learnable fusion parameter, followed by normalized residual fusion to preserve the original backbone feature representation while enhancing discriminative feature learning. The contributions of this study are as follows:

- A modified ResNet50-based architecture, named ResNet50-SCPA-Net, was proposed for liver cirrhosis stage classification using liver MRI images.
- A Parallel Attention Fusion (PAF) module integrating SE channel attention and CBAM spatial attention was incorporated into the ResNet50 backbone to enhance feature representation.
- A learnable fusion parameter (*α*) was introduced to adaptively combine the channel-wise and spatial attention features during training.
- A normalized residual fusion strategy was employed to preserve the original backbone feature representation while facilitating stable feature fusion.
- Grad-CAM visualization was used to provide visual interpretation of the model predictions.
- Comparative experiments, ablation studies, and patient-level five-fold cross-validation were conducted to evaluate the effectiveness of the proposed framework.

## 2 Methods

### 2.1 Dataset details

T2 weighted 2D MRI from the CirrMRI600 + database was used in this experiment (Jha et al., 2025). The database consisted of four classes: healthy, mild, moderate, and severe, with 53, 130, 114 and 73 instances of healthy, mild, moderate and severe liver disease, respectively, comprising a total of 370 patients. The data samples were randomly partitioned into training and test datasets in an 80:20 ratio. Table 2 shows the distribution of patients and images across the liver cirrhosis stages.

**Table 2:** Distribution of patients and images across liver cirrhosis stages.

Stage	Train patients	Test patients	Total patients	Total images
Healthy	39	14	53	2,068
Mild	109	21	130	2,854
Moderate	93	21	114	2,419
Severe	55	18	73	1,473
Total	296	74	370	8,814

### 2.2 Preprocessing

In this study, the images were rescaled to a standard dimension of 224 × 224 pixels to align with the input criteria of the ResNet50 backbone. Although MRI images are naturally grayscale, they were composed of three channels to ensure compatibility with the pretrained model in the ImageNet dataset. Augmentation was performed only on the training data to improve the learning ability of the model and to reduce overfitting. The augmentation techniques included horizontal flipping, small rotations, affine transformations, and brightness and contrast adjustment. To address the class imbalance, the class weights were calculated from the training dataset and incorporated into the weighted cross-entropy loss function during training. The weight of each class was calculated as defined in Equation 1:

$$w_{i}=\frac{N}{K*n_{i}}$$ (1)

where N denotes the total number of training samples, K represents the number of classes, and $n_{i}$ corresponds to the number of samples in class i. This weighting strategy assigns greater importance to underrepresented classes, thereby reducing the bias toward dominant classes during training. During inference, no class weighting was applied, and predictions were made based on the learned feature representations.

### 2.3 Proposed model

**Figure 1:**
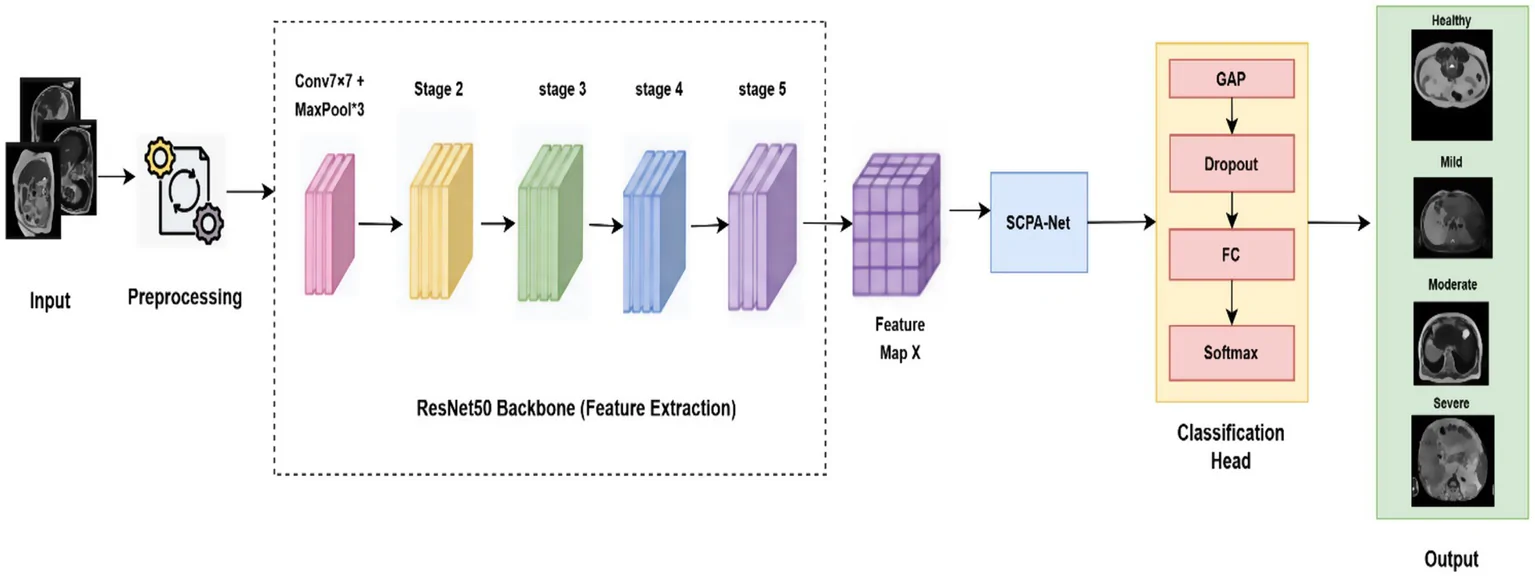
Architecture of the proposed model.

**Figure 2:**
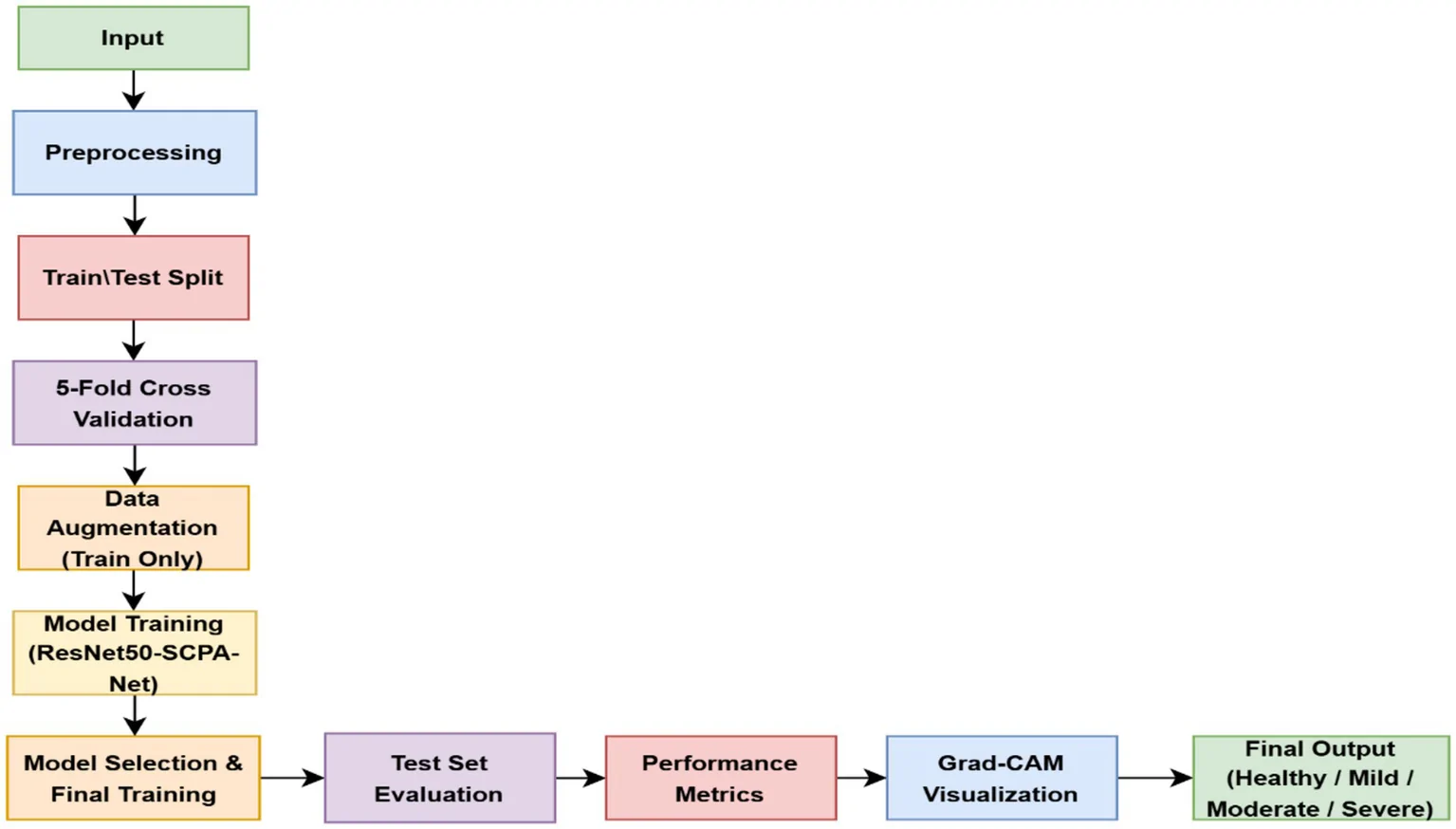
Experimental workflow.

The SE-CBAM Parallel Attention Network (SCPA-Net) was built on a ResNet50 backbone pretrained on ImageNet. The proposed framework enhances feature representation by integrating squeeze-and-excitation (SE) channel attention and Convolutional Block Attention Module (CBAM) spatial attention in a parallel configuration, referred to as the Parallel Attention Fusion (PAF) module. Unlike conventional sequential attention mechanisms, the proposed architecture processes channel and spatial attention simultaneously on the same backbone feature maps and adaptively fuses their outputs through a learnable gating parameter to improve discriminative feature learning. Figure 1 illustrates the overall architecture of the proposed ResNet50-SCPA-Net. Figure 2 illustrates the experimental workflow of the proposed model framework.

The input MRI images were initially preprocessed by resizing and normalization, followed by a train–test split. The training data are further divided using 5-fold cross-validation, and data augmentation was applied to enhance generalization. The model was then trained using the SCPA-Net architecture, and the best-performing model was selected for final training. The trained model was evaluated on the test set using standard performance metrics. Grad-CAM visualization is used to interpret the model predictions, and the output was categorized into four classes: healthy, mild, moderate, and severe.

The proposed SCPA-Net follows the standard ResNet50 architecture, consisting of an initial convolutional layer followed by four residual stages arranged in a 3–4–6–3 bottleneck configuration (He et al., 2016). After the final feature extraction stage for ResNet50, the extracted feature representation was refined using the proposed Parallel Attention Fusion (PAF) module, which combines the SE channel attention and CBAM spatial attention in a parallel configuration. The proposed attention mechanism enhances the channel-wise and spatially informative features associated with the progression of liver cirrhosis. After attention refinement, global average pooling (GAP) was applied, followed by a fully connected (FC) layer and softmax classifier for predicting the stage of liver cirrhosis.

### 2.4 PAF module

**Figure 3:**
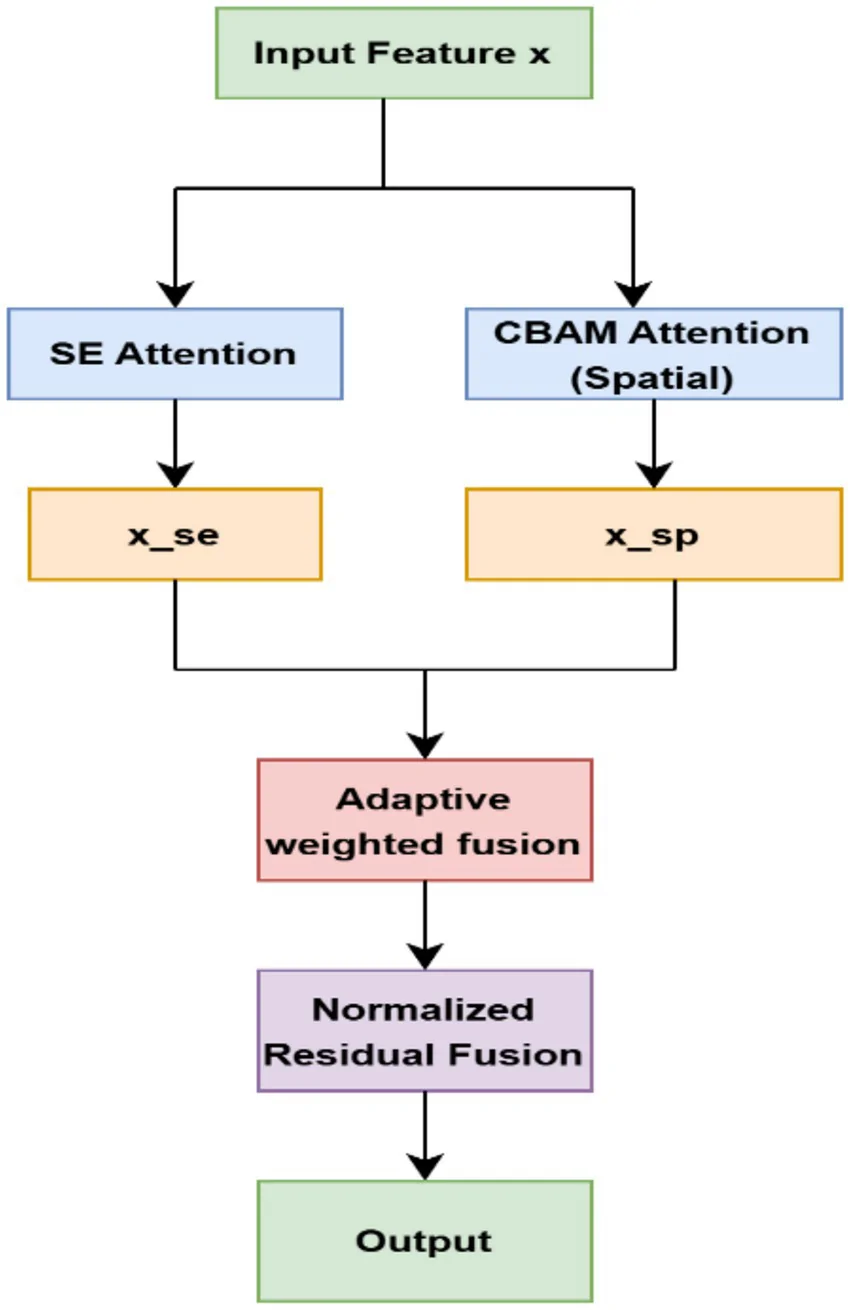
PAF model.

Figure 3 illustrates the proposed PAF module design. The input feature map $X\in {\mathbb{R}}^{B\times 2048\times H\times W}$ , obtained from the final stage of ResNet50, passes through two attention branches that operate simultaneously: the SE channel attention branch and CBAM spatial attention branch. The outputs of both attention branches are adaptively fused using learnable gating parameters. Subsequently, the fused feature representation is combined with the original feature map through normalized residual fusion to generate the final output feature representation. Let X denote the input feature map of the PAF module.

### 2.5 SE channel attention

The SE attention mechanism (Hu et al., 2018) performs adaptive channel-wise feature recalibration by applying global average pooling to generate a channel descriptor as defined in Equation (2):

$$z_{c}=(\frac{1}{H\times W})\sum_{i=1}^{H}\sum_{j=1}^{W}X_{c(i,j)}$$ (2)

The attention weights for the channels are calculated using two fully connected layers with ReLU and sigmoid activation functions as defined in Equation 3:

$$s=\sigma (W_{2}\cdot \text{ReLU}(W_{1}\cdot z))$$ (3)

The channel-refined feature map is obtained through channel-wise scaling:

$$X_{\left\{\mathrm{SE}\right\}}=s⊗X$$ (4)

Equation 4, where *σ* is the sigmoid activation function, and ⊗ is element-wise multiplication. The reduction ratio r was fixed at 16, resulting in a bottleneck mid-dimension of 128 pixels. The SE branch refines the feature representation by learning the relative importance of individual channels, thereby enhancing the clinically significant feature patterns associated with cirrhosis severity and suppressing redundant responses.

### 2.6 CBAM spatial attention

The CBAM spatial attention mechanism (Woo et al., 2018) focuses on identifying informative spatial regions within the feature map through a deeper two-stage convolutional pathway without batch normalization. Channel-wise average pooling and max pooling were first applied across the channel dimension of X and concatenated to form a two-channel spatial descriptor as defined in Equation (5):

$$f=[\text{AvgPoo}l_{\left\{\text{channel}\}(X\right)};\text{MaxPoo}l_{\left\{\text{channel}\}(X\right)}]$$ (5)

The concatenated spatial descriptor is processed using a 3×3 convolution that expands the feature representation from 2 to 16 channels, followed by a ReLU activation function as as defined in Equation (6):

$$f_{\mathrm{mid}}=\text{ReLU}(\mathrm{Con}v_{\left(3\times 3)(f,2\to 16\right)})$$ (6)

Subsequently, a 7×7 convolution reduces the feature representation from 16 to 1 channel, followed by a sigmoid activation function to produce the spatial attention map as defined in Equation (7):

$$M_{s}=\sigma (\mathrm{Con}v_{\left(7\times 7)(f_{\mathrm{mid}},16\to 1\right)})$$ (7)

The spatially refined feature representation is computed as defined in Equation (8):

$$X_{\mathrm{SP}}=M_{s}⊗X$$ (8)

This deeper spatial pathway enables the network to learn richer cross-channel spatial interactions, allowing it to focus on structural liver tissue changes associated with cirrhosis progression while suppressing the responses from uninformative spatial regions.

### 2.7 Parallel fusion strategy

A single learnable scalar gating parameter *α* was introduced to adaptively combine the channel and spatial attention outputs. The parameter is represented in the logit space and normalized using a sigmoid activation function as defined in Equation (9):

$$\alpha =\sigma (\alpha_{\text{logit}})$$ (9)

The parameter α is initialized with $\alpha_{\text{logit}}=0.0$ , corresponding to α=0.5 at the beginning of training, thereby ensuring an equal contribution from both attention branches during initialization (Algorithm 1). Adaptive weighted fusion is computed as defined in Equation (10):

$$A_{\text{fused}}=\alpha X_{\mathrm{SE}}+(1-\alpha )X_{\mathrm{SP}}$$ (10)

Algorithm 1 Parallel attention fusion (PAF) module.
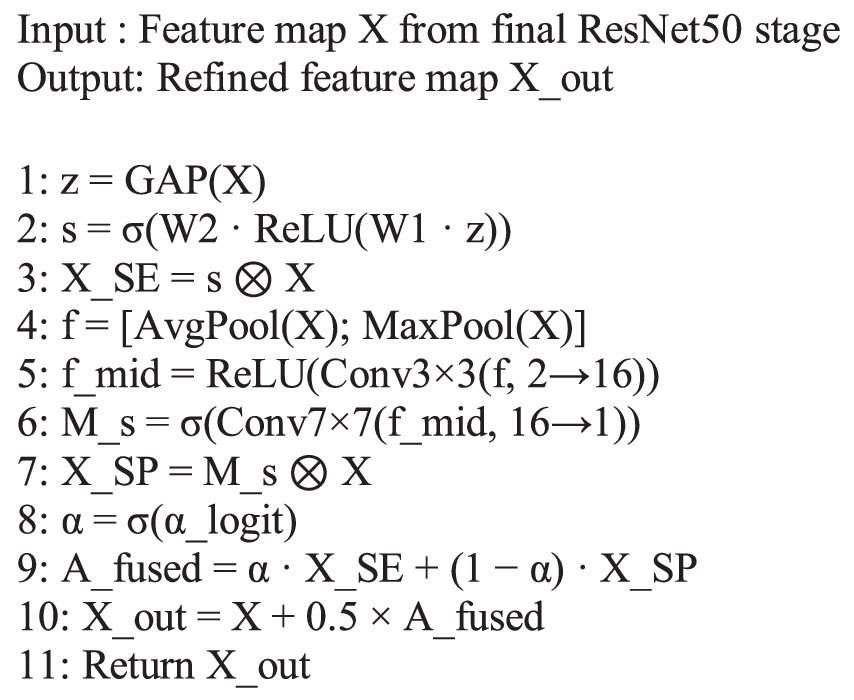

where (1α) represents the contribution of the spatial attention branch to the final score. When α→1 , the model gives greater importance to channel-wise feature recalibration through the SE branch, whereas when α→0 , the model emphasizes spatial feature localization through the CBAM spatial branch. To preserve the residual learning behavior of the backbone network and prevent excessive amplification of the attention responses, normalized residual fusion is applied as defined in Equation (11):

$$X_{\text{out}}=X+0.5\times A_{\text{fused}}$$ (11)

The scaling factor of 0.5 constrains the contribution of the fused attention representation to the scale of the backbone residual feature map, thereby improving the optimization stability and reducing the risk of attention explosion during training. Unlike sequential attention mechanisms, the proposed parallel fusion strategy models complementary channel dependencies and spatial feature representations. The proposed framework effectively exploits both global channel and spatial contextual information, thereby improving the discriminative feature learning for liver cirrhosis stage classification. Table 3 presents the configuration of the proposed model. All experiments were performed on an NVIDIA Tesla T4 GPU via Google Colab, with mixed precision being applied to decrease GPU consumption. Algorithm 1 summarizes the workflow of the proposed Parallel Fusion method.

**Table 3:** Training setup.

Parameter	Parameter
Backbone	ResNet-50
Input Size	224 × 224
Batch Size	16
Epochs	30
Optimizer	AdamW
Learning Rate	3 × 10–5
Weight Decay	1 × 10–4
Loss Function	Weighted Cross-Entropy
Classifier	Dropout 0.3
Cross-Validation	5-Fold
Mixed Precision	Enabled

### 2.8 Evaluation metrics

To assess the performance of the model, parameters such as accuracy, balanced accuracy, recall, precision, F1 score, and area under the curve (AUC) were considered. Accuracy represents the degree of correctness of the classifier based on the ratio of the number of correctly classified instances to the total number of instances in the dataset as defined in Equation (12).

Accuracy=(TP+TN)/(TP+TN+FP+FN (12)

Balanced accuracy considers class imbalance by computing the mean recall achieved in each class. Sensitivity is the true positive rate, and specificity is the true negative rate as defined in Equation (13).

Balanced\Accuracy=(Sensitivity+Specificity)/2 (13)

Recall was used to measure the performance of the model in classifying positive samples. A high recall indicates low false negatives, which is important for medical purposes as defined in Equation 14.

Recall=TP/(TP+FN) (14)

Precision is an evaluation measure used to assess the correctness of the positive prediction made by the model. A high level of precision indicates a low rate of false positives as defined in Equation (15).

Precision=TP/(TP+FP) (15)

The F1-Score is the harmonic mean of Precision and Recall, thus balancing both. This measure is especially effective for imbalanced classes as defined in Equation (16).

F1−score=2×(Precision×Recall)/(Precision+Recall) (16)

## 3 Results and discussion

This section presents the performance of the proposed ResNet50-SCPA-Net model for liver cirrhosis stage classification. The results were compared with those of commonly used CNN architectures, including VGG16, DenseNet121, ResNet50, and EfficientNet-B0, across four classes: healthy, mild, moderate, and severe. Table 4 compares the proposed ResNet50-SCPA-Net model with the baseline architectures, including DenseNet121, EfficientNetB0, and VGG16. The proposed model achieved an accuracy of 80.82%, balanced accuracy of 81.93%, F1-score of 82.55%, and AUC of 93.05%.

**Table 4:** Performance comparison of ResNet50-SCPA-Net with baseline models.

Model	Accuracy (%)	95% confidence interval	Balanced accuracy (%)	F1-score (%)	AUC (%)
Proposed (ResNet50-SCPA-Net)	80.82	70.34–88.22	81.93	82.55	93.05
VGG16	69.86	58.56–79.18	71.85	72.42	88.83
DenseNet121	72.20	61.44–81.51	73.32	73.85	89.98
EfficientNetB0	71.23	59.99–80.35	72.04	73.66	89.75

**Figure 4:**
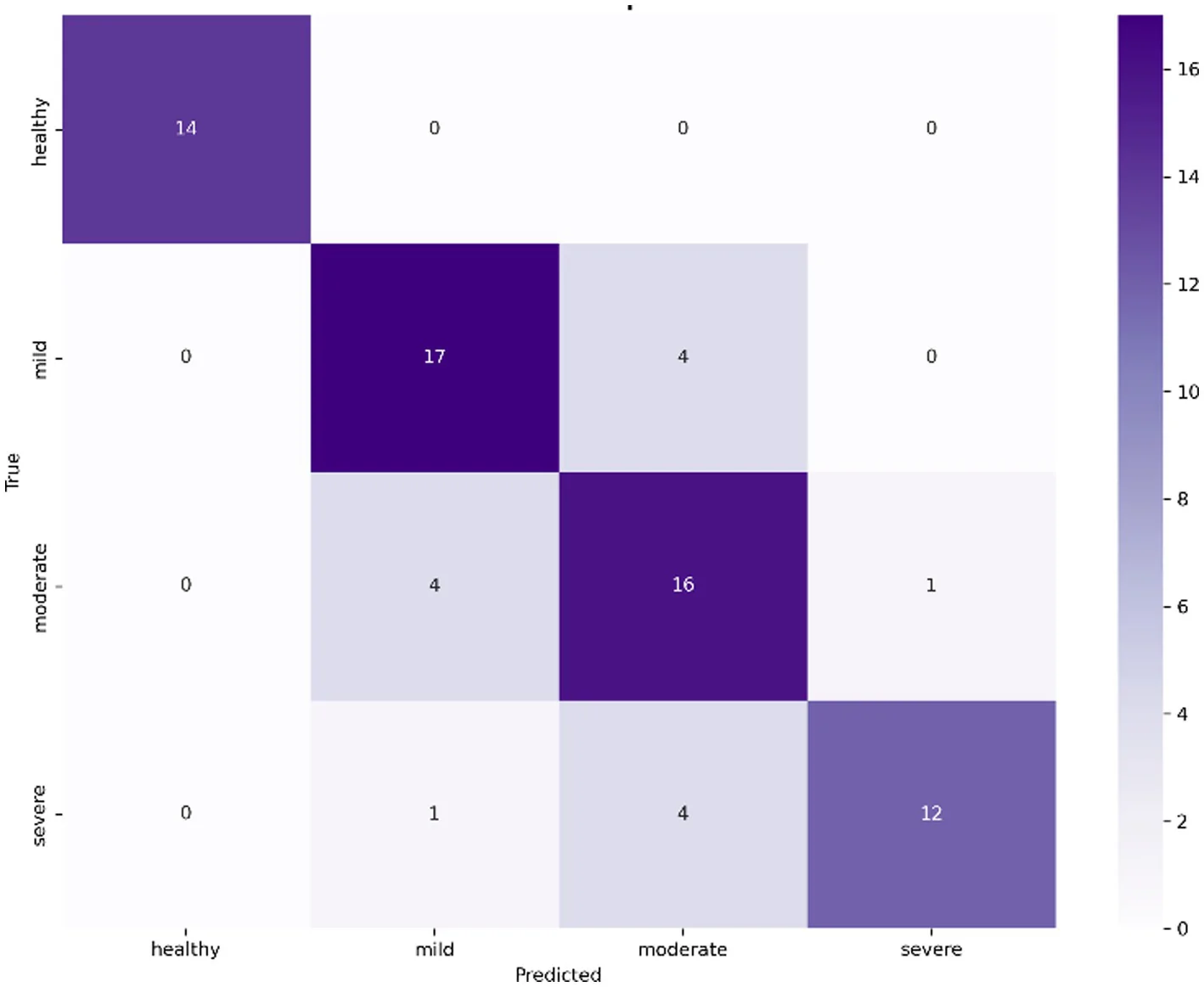
Confusion matrix for ResNet50-SCPA-Net.

**Figure 5:**
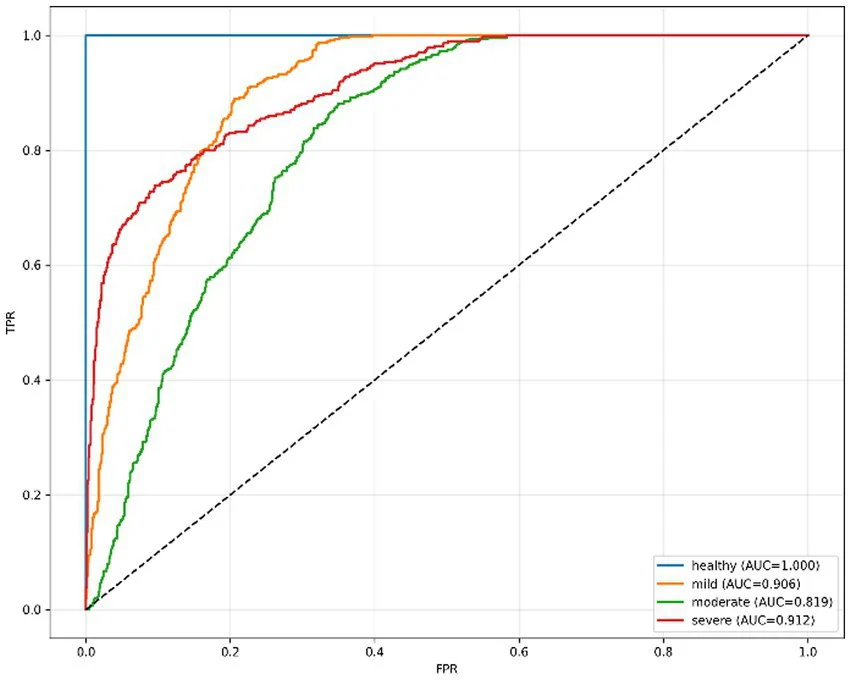
ROC curve.

The results indicate that the proposed parallel attention fusion strategy is effective in capturing both channel-wise and spatial feature representations for liver cirrhosis staging classification. Overall, the proposed model demonstrated competitive performance compared to the baseline architectures. Figure 4 shows the confusion matrix of the proposed model for multiclass liver cirrhosis stage classification. The diagonal entries represent the correctly classified samples, whereas the off-diagonal entries indicate the misclassifications. All 14 healthy samples were classified accurately. In the mild class, 17 samples were correctly identified, whereas four samples were misclassified as moderate. For the moderate class, 16 samples were correctly classified, with 4 misclassified as mild and 1 as severe. In the severe class, 12 samples were correctly classified, whereas four and one samples were misclassified as moderate and mild, respectively. Figure 5 shows the ROC curves of the proposed ResNet50-SCPA-Net model for liver cirrhosis staging. The healthy class achieved the highest AUC value of 1.000, followed by the severe (0.912) and mild (0.906) classes. The moderate class achieved an AUC of 0.819. The results indicate that the proposed model achieved good classification performance across all classes. Table 5 presents a class-wise performance comparison of the proposed model and baseline architectures across different liver cirrhosis stages. All models achieved 100% classification accuracy for the healthy class. For the mild class, the proposed model achieved an accuracy of 80.95%. For the moderate class, the proposed model achieved an accuracy of 76.19%. For the severe class, the proposed model achieved an accuracy of 70.59%. These results indicate that the performance varied across classes, with relatively lower values observed for the moderate and severe stages for all models. Table 6 presents the ablation study results for the different attention configurations for the liver cirrhosis stage classification. The accuracy, balanced accuracy, F1 score, and AUC values obtained by the baseline ResNet50 model were 73.15, 74.40, 75.75, and 89.18%, respectively. The accuracy, balanced accuracy, F1 score, and AUC values obtained by the ResNet50 + SE model were 68.49, 70.66, 69.49, and 90.83%, respectively.

**Table 5:** Class-wise performance comparison across liver cirrhosis stages.

Stage	Proposed (ResNet50-SCPA-Net)	VGG16	DenseNet121	EfficientNetB0
Healthy	100%	100%	100%	100%
Mild	80.95%	76.19%	80.95%	80.95%
Moderate	76.19%	52.38%	49.62%	52.38%
Severe	70.59%	58.82%	62.45%	58.82%

**Table 6:** Ablation study of the proposed ResNet50-SCPA-Net model.

Model	Accuracy (%)	Balanced accuracy (%)	F1-score (%)	AUC (%)
ResNet50	73.15	74.40	75.75	89.18
ResNet50 + SE	68.49	70.66	69.49	90.83
ResNet50 + CBAM	72.60	73.95	73.73	91.68
ResNet50 + SE + CBAM	75.34	76.89	77.40	91.53
ResNet50 + SE + Spatial CBAM (Sequential)	76.71	78.08	78.39	92.07
Proposed (ResNet50-SCPA-Net)	80.82	81.93	82.55	93.05

The accuracy, balanced accuracy, F1-score, and AUC values obtained using the ResNet50 + CBAM model were 72.60, 73.95, 73.73, and 91.68%, respectively. Combining the SE and CBAM modules resulted in the ResNet50 + SE + CBAM model, which achieved an accuracy of 75.34%, a balanced accuracy of 76.89%, an F1-score of 77.40%, and an AUC of 91.53%. The ResNet50 + SE + Spatial CBAM (Sequential) model achieved an accuracy of 76.71%, a balanced accuracy of 78.08%, an F1-score of 78.39%, and an AUC of 92.07%. The proposed ResNet50-SCPA-Net achieved the highest performance, with an accuracy of 80.82%, a balanced accuracy of 81.93%, an F1-score of 82.55%, and an AUC of 93.05%. These results indicate that the proposed parallel attention fusion strategy provides improved classification performance compared with the baseline model and the other attention-based configurations. Table 7 shows the 5-fold cross-validation results of the proposed ResNet50-SCPA-Net model for staging liver cirrhosis. The accuracy values obtained for folds 1–5 were 70.22, 71.41, 72.67, 71.68, and 71.70%, respectively. The balanced accuracy values were 73.35, 73.05, 73.74, 74.28, and 73.41%. Similarly, the F1-score values obtained for the five folds were 72.50, 72.37, 72.83, 74.09, and 73.83%. The overall mean accuracy achieved by the model was 71.54% ± 0.01. The mean balanced accuracy and mean F1-score were 73.57% ± 0.004 and 73.13% ± 0.01, respectively. The entire dataset was initially divided into 80% training data and 20% independent test data. During model development, five-fold cross-validation was performed exclusively on the 80% training set, where each fold used different training and validation partitions. The reported cross-validation accuracy (71.54%) represents the average validation accuracy across the five folds. After selecting the optimal model configuration, the network was retrained using the entire 80% training set and subsequently evaluated on the independent 20% hold-out test set, yielding a test accuracy of 80.82%.

**Table 7:** 5-fold cross-validation results of the proposed ResNet50-SCPA-Net model.

Fold	Accuracy (%)	Balanced accuracy (%)	F1-score (%)
1	70.22	73.35	72.50
2	71.41	73.05	72.37
3	72.67	73.74	72.83
4	71.68	74.28	74.09
5	71.70	73.41	73.83
Overall (Mean ± Std)	71.54 ± 0.01	73.57 ± 0.004	73.13 ± 0.01

**Figure 6:**
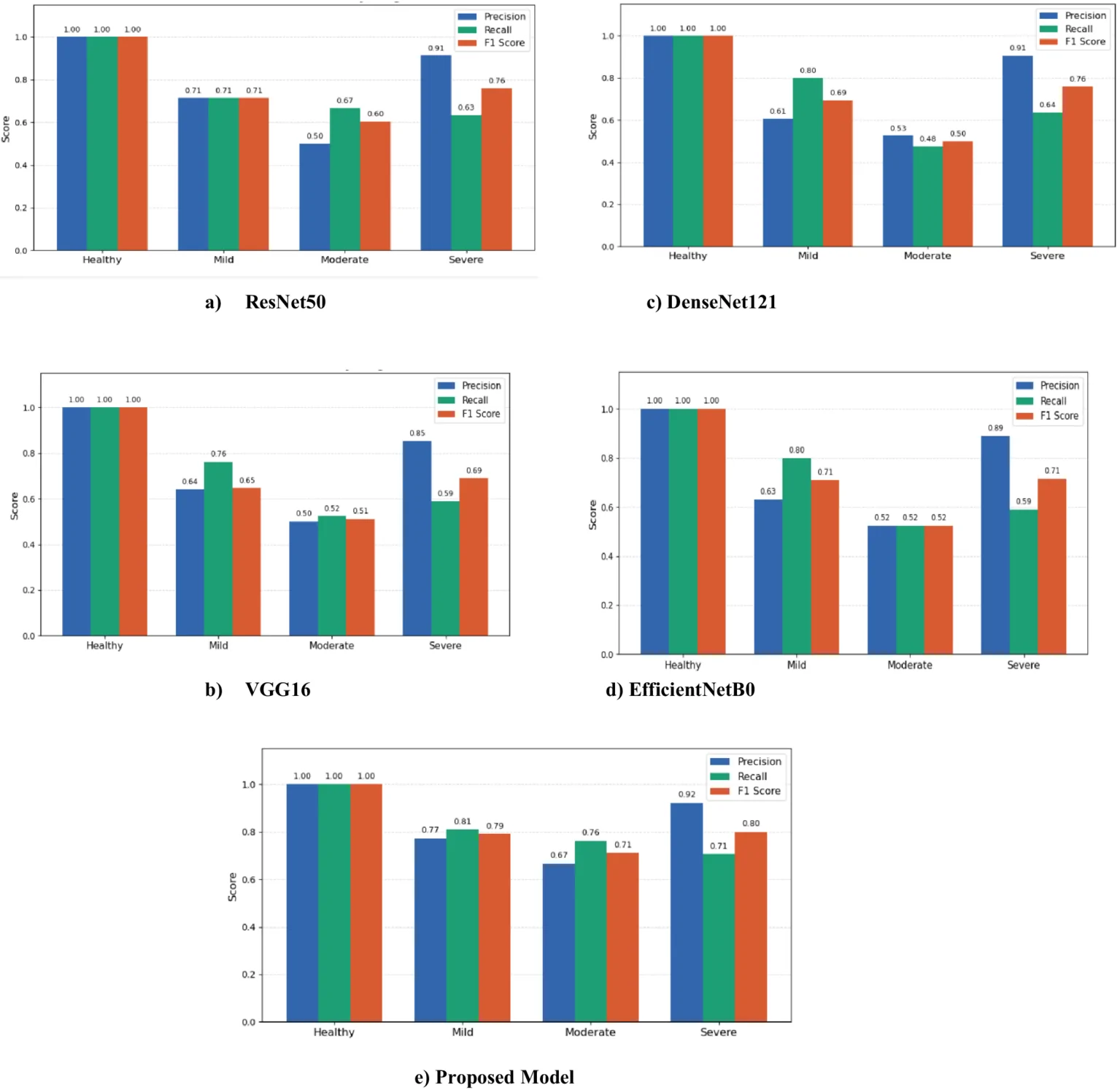
Class-wise performance comparison of **(a)** ResNet50, **(b)** VGG16, **(c)** DenseNet121, **(d)** EfficientNetB0, and **(e)** the proposed model.

Figure 6 shows the class-wise precision, recall, and F1-score for the baseline and proposed models across different liver cirrhosis stages. For the healthy class, all models achieved perfect performance, with precision, recall, and F1-score values of 1.0. This indicates that healthy samples are clearly distinguishable from the diseased classes in the dataset. In the mild class, the performance varied across models, with moderate differences observed in recall and F1-score. For the moderate class, comparatively lower values were observed across all models, suggesting increased difficulty in distinguishing this stage from the others. For the severe class, the models show relatively higher precision, whereas the recall and F1-score varied across different architectures. The proposed model showed comparable performance across all classes, with relatively balanced precision, recall, and F1-score values compared with the baseline models. The ablation results indicate that different attention configurations contribute differently to feature learning. Although the sequential combination of SE and spatial CBAM improved the classification performance compared with the individual attention modules, the proposed Parallel Attention Fusion (PAF) strategy achieved the best overall performance. Unlike the sequential attention configuration, the proposed approach integrates SE channel attention and CBAM spatial attention in parallel through a learnable fusion parameter, enabling complementary channel-wise and spatial feature representations to be combined while preserving the original feature information through normalized residual fusion. These findings suggest that the proposed parallel fusion strategy provides a more effective integration of channel-wise and spatial attention than the evaluated sequential attention configuration.

**Figure 7:**
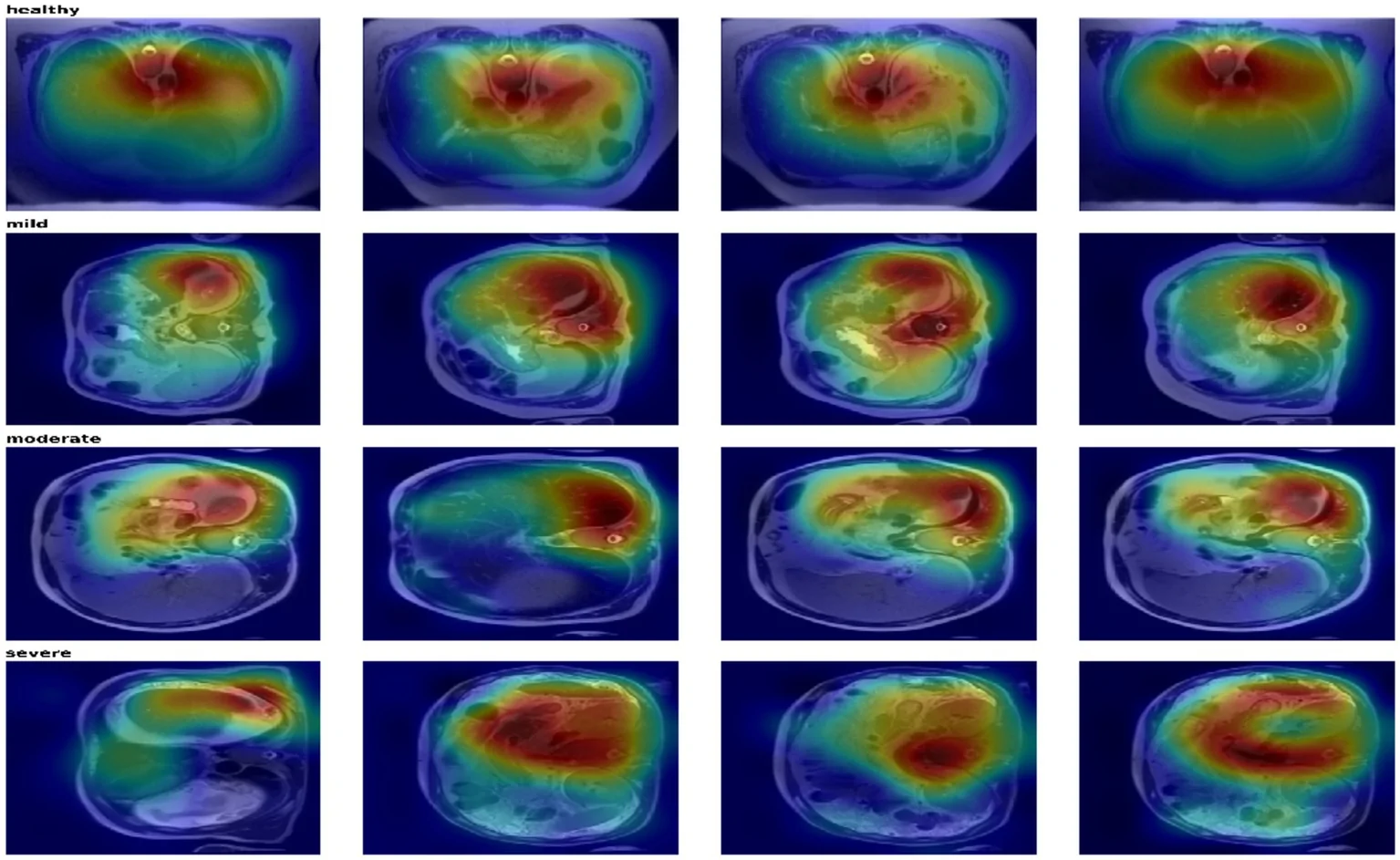
Grad-CAM visualization.

Table 8 presents the McNemar’s test results for the proposed ResNet50-SCPA-Net and the baseline models. Statistically significant differences were observed between the proposed model and DenseNet121 (x^2^ = 5.421, *p* = 0.0199), EfficientNet-B0 (x^2^ = 4.876, *p* = 0.0273), ResNet50 (x^2^ = 8.312, *p* = 0.0039), and VGG16 (x^2^ = 5.214, *p* = 0.0224). For all comparisons, the *p*-values were less than the significance level of 0.05, indicating statistically significant differences in the classification results between the proposed model and the corresponding baseline models. The adaptive fusion parameter was initially set to provide equal contributions from the SE and spatial attention branches during training. After training, the obtained fusion weights were 0.49 and 0.51 for the SE and spatial attention, respectively. These values indicate that both attention branches contributed almost equally during feature refinement. Table 9 presents a comparison of the computational complexity, model parameters, FLOPs, and inference time of the proposed ResNet50-SCPA-Net with four baseline models: ResNet50, VGG16, DenseNet121, and EfficientNetB0. The proposed model achieved the highest classification accuracy of 80.82% (95% CI: 70.34–88.22). Compared with the baseline ResNet50, the proposed model required only a marginal increase in the number of parameters (24.042 M vs. 23.516 M), computational cost (8.265 vs. 8.263 GFLOPs), and inference time (6.276 vs. 5.814 ms). DenseNet121 and EfficientNetB0 required fewer parameters and lower computational costs than the proposed model but achieved lower classification accuracy. Although DenseNet121 had relatively fewer parameters and lower FLOPs, it exhibited the highest inference time (13.591 ms) among the evaluated models. VGG16 exhibited the highest computational cost (30.693 GFLOPs) among all the evaluated models. Figure 7 shows the Grad-CAM visualizations generated by the proposed ResNet50-SCPA-Net model for the healthy, mild, moderate, and severe stages of liver cirrhosis. The highlighted regions indicate the areas that contributed most to the model’s classification decisions. The Grad-CAM results indicated that the model primarily focused on the liver region during prediction, suggesting that the classification was based on anatomically relevant features rather than the surrounding tissues. The highlighted regions were generally consistent with areas where liver cirrhosis may result in structural changes. This study had some limitations. First, the model was evaluated using a limited public dataset, which may have affected the generalizability of our results. Second, the experiments were conducted using data from a single imaging source, and external validation using independent multicenter datasets was not performed in this study. In addition, the study focused only on liver MRI images, and the performance of the proposed model on other imaging modalities was not evaluated.

**Table 8:** McNemar’s test results comparing the proposed ResNet50-SCPA-Net with baseline models.

Comparison	McNemar statistic (x^2^)	*p*-value	Significant (α = 0.05)
Proposed Model vs. DenseNet121	5.421	0.0199	Yes
Proposed Model vs. EfficientNet-B0	4.876	0.0273	Yes
Proposed Model vs. ResNet50	8.312	0.0039	Yes
Proposed Model vs. VGG16	5.214	0.0224	Yes

**Table 9:** Comparison of the proposed ResNet50-SCPA-Net and baseline models in terms of performance and computational complexity.

Model	Accuracy (%)	95% confidence interval	Params (M)	FLOPs (G)	Inference time (ms)
Proposed (ResNet50-SCPA-Net)	80.82	70.34–88.22	24.042	8.265	6.276
VGG16	69.86	58.56–79.18	14.72	30.693	7.966
DenseNet121	72.20	61.44–81.51	7.98	5.279	13.591
EfficientNetB0	71.23	59.99–80.35	4.012	0.769	7.860
ResNet50	73.15	62.44–81.51	23.516	8.263	5.814

## 4 Conclusion

In this study, a modified ResNet50-based model, called ResNet50-SCPA-Net, was developed for liver cirrhosis stage classification using liver MRI images. The model integrated SE channel attention and CBAM spatial attention through the Parallel Attention Fusion (PAF) module, enabling complementary refinement of channel-wise and spatial features using a learnable fusion parameter. It achieved an accuracy of 80.82%, a balanced accuracy of 81.93%, an F1-score of 82.55%, and an AUC of 93.05%. In addition, 5-fold cross-validation was performed to evaluate the robustness of the model across different data splits. Grad-CAM visualization was further applied to identify the image regions that contributed to the prediction process. Overall, the proposed approach demonstrated its potential for the automated classification of liver cirrhosis stages from MRI images. In future studies, the model can be evaluated using larger multicenter datasets and different imaging modalities to further investigate its generalizability and performance.

## Data Availability

The dataset used in this study is publicly available at https://osf.io/cuk24/.
